# Regulatory B Cells: Role in Type 1 Diabetes

**DOI:** 10.3389/fimmu.2021.746187

**Published:** 2021-09-20

**Authors:** Joanne Boldison, F. Susan Wong

**Affiliations:** ^1^Institute of Biomedical & Clinical Science, University of Exeter, Exeter, United Kingdom; ^2^Division of Infection & Immunity, School of Medicine, Cardiff University, Cardiff, United Kingdom

**Keywords:** IL-10, B cell, type 1 diabetes, frequency, function

## Abstract

Regulatory B cells (Bregs) have an anti-inflammatory role and can suppress autoimmunity, by employing both cytokine secretion and cell-contact mediated mechanisms. Numerous Breg subsets have been described and have overlapping phenotypes in terms of their immune expression markers or cytokine production. A hallmark feature of Bregs is the secretion of IL-10, although IL-35 and TGFβ−producing B cells have also been identified. To date, few reports have identified an impaired frequency or function of Bregs in individuals with type 1 diabetes; thus our understanding of the role played by these Breg subsets in the pathogenesis of this condition is limited. In this review we will focus on how regulatory B cells are altered in the development of type 1 diabetes, highlighting both frequency and function and discuss both human and animal studies.

## Introduction

It is now well-established that regulatory B cells (Bregs) can dampen immune responses and play a role in maintaining immune tolerance. These immunosuppressive Bregs are generally named for the anti-inflammatory cytokines that they produce to exert their regulatory effects, and so a variety of Bregs have been identified. The cytokine most widely associated with Bregs is Interleukin-(IL-)10 (1) and thus has been the major focus of many studies into the failure of Bregs to suppress inflammation in autoimmune conditions. IL-10 independent mechanisms have been identified, including suppression mediated by contact of cell surface molecules (2, 3) or other soluble mediators such as the production of TGFβ (4) and IL-35 (5). However, currently there are no reports of alterations in these IL-10 independent regulatory B cell populations, either in number or function, in human type 1 diabetes; thus their contribution to type 1 diabetes remains an outstanding question.

In type 1 diabetes B cells are typically understood to play a pathogenic role in disease, likely through the production of inflammatory cytokines and presentation of autoantigens to T cells (6). This has been emphasized by the use of Rituximab in clinical trials and the observed temporary delay in the loss of C-peptide (7). However, studies of other autoimmune diseases have highlighted the essential role for regulatory B cells (8) and this has now been reflected in type 1 diabetes, although comparatively with fewer studies. Regulatory B cells in other autoimmune diseases, including diabetes, has recently been reviewed (9). It is imperative that we further understand the balance between effector and regulatory B cells in order to improve immunotherapeutic treatments targeting these lymphocytes, including utilizing Bregs as a therapeutic option. This review will focus on the emerging literature on Bregs and discuss their role in type 1 diabetes.

## Regulatory B Cell Phenotypes

Studies in both human and mouse have contributed to identifying numerous IL-10-producing Breg subsets using a variety of immune markers, some of which overlap, to indicate a regulatory population. In humans, several Breg subsets enriched at different stages of B cell maturation, including immature B cells (CD24^hi^CD38^hi^) (10), memory B cells (CD24^hi^CD27^+^ [B10]) (11) and plasmablasts (CD27^int^CD38^+^) (12) have been identified. Similarly, in mice, various subsets have been identified in the transitional (13) and marginal zone (14) B cell compartments, including specific mouse subsets that parallel human B10 cells (11, 15) and human plasmablasts (12).

Other human regulatory B cell subsets have also been described including CD19^+^Tim-1^+^ B cells (16) and CD39^+^CD73^+^ Bregs (17), with equivalent subsets described in mice (18, 19). In addition, human CD25^hi^CD71^hi^ B cells produce IgG4 and are designated as regulatory Br1 cells (20). However, these subsets have not yet been described in human type 1 diabetes. The diversity and identification of Breg phenotypes has been reviewed extensively (9, 21, 22). The range and variability in methods which induce IL-10-producing B cells, along with a lack of a key definitive marker, makes it difficult to define a Breg cell without assessing IL-10 production, as a key function. Therefore, the evaluation of IL-10-production during the differentiation and developmental stages of B cells is important, as demonstrated by Iwata et al. reporting the distinction between B10 cells and B10-progenitor cells (B10_PRO_) (11). The different subsets of Bregs that have been assessed, specifically in studies of type 1 diabetes, is discussed (in *Impaired Regulatory B Cell Mechanisms in type 1 diabetes*) and **Table 1**.

**Table 1:** Evidence for numerical defects in Bregs in type 1 diabetes.

Study	Phenotype of B cell	Change in cell frequency (*vs.* healthy donors)	Stimulus for IL-10 induction	Diabetes duration (years)	Age of donors with diabetes (years)	Age of healthy donors (years)
De Filippo., et al. (23)	CD5^+^CD19^+^	Increase (median 250 *vs.* 95 [cells mm^3^])*	NM	<30days diagnosis	Mean ±SD: 6.7±2.5	Age-matched
Deng., et al. (24)	CD19^+^CD5^+^CD1d^hi^ (B10 cells)	Decrease (Median, values not described, [B10% of CD19^+^]***	NM	Mean ±SD: 3.1 ± 3.5	Mean ±SD: 28.53 ±16.21	Mean ±SD: 41.37 ± 13.52
Habib., et al. (25)	CD19^+^CD27^-^CD10^+^CD24^hi^CD38^hi^	Increase (Mean, values not described, [%transitional/CD19^+^]*	NM	Not reported	Range: 19-36	Range: 19-46
Hanley., et al. (26)	CD24^hi^CD38^hi^	Decrease (Mean ±SD: 1.54± 0.85 *vs.* 2.67 ±1.15 [% of CD19^+^]**	NM	Mean ±SD: 19.25 ± 10.99	Mean ±SD: 34.75 ± 13.13	Mean ±SD: 31.75 ±8.17
Thompson., et al. (27)	CD19^+^CD27^-^CD24^hi^CD38^hi^ (transitional)	No difference (*P*=0.50)	NM	Range: 0.2-31. Median: 1.8	Range: 9-42. Median: 20	Range:18-37. Median: 27
	IL-10^+^ B cells	No difference (*P*=0.74)	Anti-CD40 + IL-21 (3 days) + CpG + LPS (last 5hrs)			
Kleffel., et al. (28)	CD19^+^IL-10^+^ B cells	Decreased (Mean ±SEM, values not described, [IL-10%]**	CD40L + LPS (4 days)	Mean ±SEM: 35 ±2.4	Mean ±SEM: 53.2 ± 2.3	Mean ±SEM: 32.1 ± 2.2
Saxena., et al. (29)	CD5^+^IL-10^+^ B cells	No difference (*P*=0.31)	PMA/Ionomycin	Range: 1.5-31.5	Range: 18-49.2	Range: 19.2-46
Wang., et al. (30)	CD24^hi^CD38^hi^	Decreased (Mean ±SEM, 5.6 ± 3.5 *vs.* 6.9 ± 3.3 [%])*	NM	Mean ±SEM 5.38± 0.72	23.76± 5.89^§^ (Range 7-29)	24.91± 2.92^§^ (Range 20-30)
	CD24^hi^CD38^hi^IL-10^+^	Decreased (Mean ±SEM, values not described, [IL-10%])***	CD40L + CpG (3 days)			
El-Mokhtar., et al. (31)	CD24^hi^CD38^hi^IL-10^+^ CD24^+^CD27^+^IL-10^+^	Decreased (% CD24^hi^CD38^hi^IL-10^+^, Mean ±SEM, 0.48 ± 0.54 *vs.* 1.3 ± 0.57)*** (% CD24^+^CD27^+^IL-10^+^, Mean ±SEM, 0.49 ± 0.57 *vs.* 1.3 ± 0.53)***	PMA/Ionomycin	Range 0.1-4.85, Median 1.6	Range 3.4-11, Median 7	Range 2.6-8.5, Median 7

All studies measured IL-10 production by intracytoplasmic staining. NM (not measured). Versus and compared to healthy donors. All studies performed in human peripheral blood. *p < 0.05, **p < 0.01, ***p < 0.001. ^§^Average age, SEM or SD not stated.

## Breg Induction and Type 1 Diabetes

**Figure 1:**
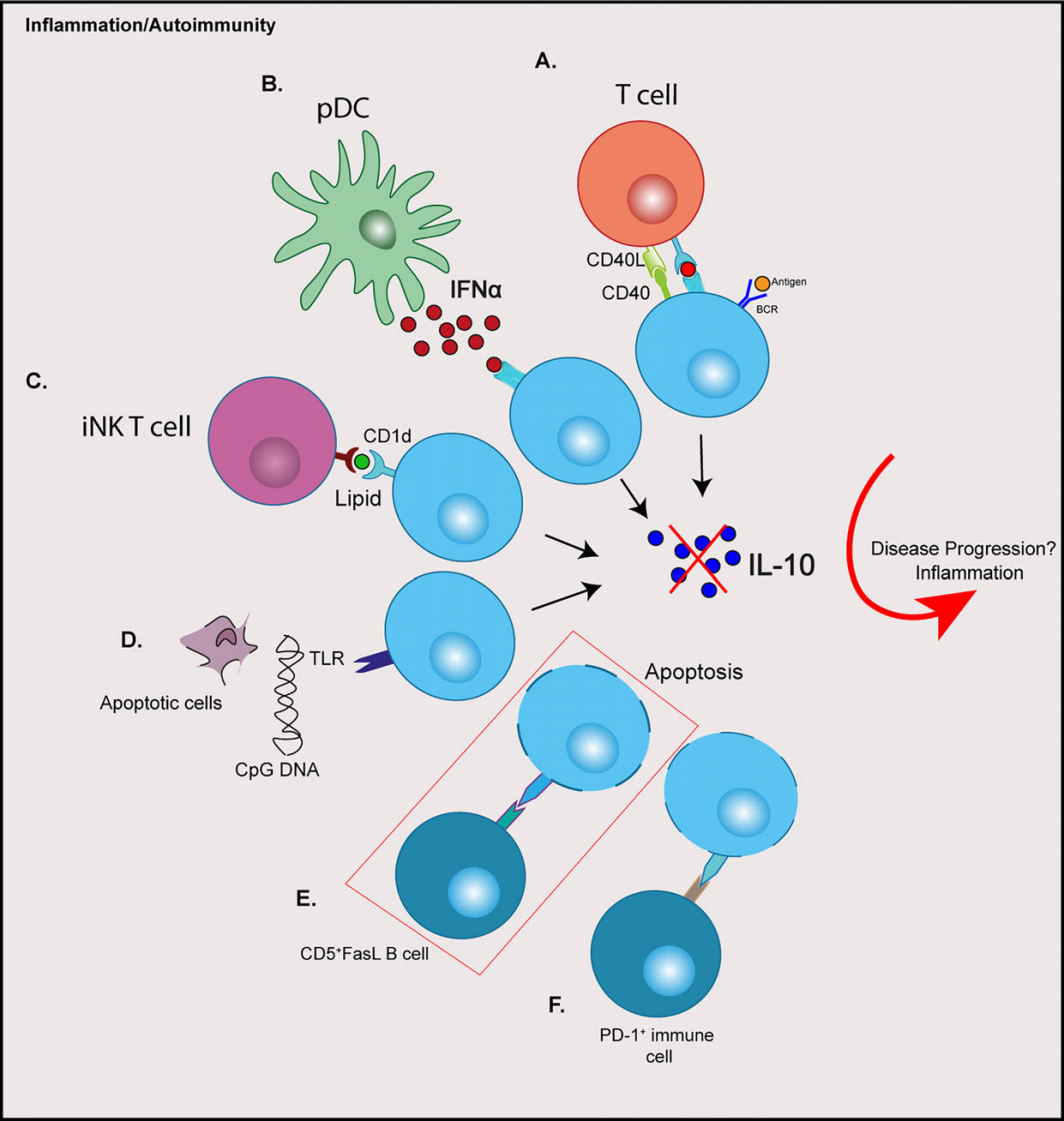
Possible contributions of immune cell crosstalk resulting in dysregulation of regulatory B cells in type 1 diabetes. **(A)** Aberrant CD40:CD40L signalling through T cells **(B)** Elevated IFNα production from pDCs **(C)** Altered iNK T cells and CD1d expression on B cells **(D)** TLR signalling from apoptotic cell debris or the presence of viruses or microbes **(E)** Increased expression of Fas on IL-10^+^ B cells are targeted by CD5^+^FasL B cells **(F)** PD-L1: PD-1 engagement resulting in increased Breg apoptosis. Red box depicts a possible mechanism reported in type 1 diabetes. CPG, cytosine-phosphate-guanine; BCR, B cell receptor; IFN, Interferon; iNK, invariant natural killer; pDCs, plasmacytoid dendritic cells; TLR, toll-like receptor; FasL, Fas-ligand; PD-L1, programmed death-ligand 1; PD-1, programmed cell death protein 1.

The heterogeneity of Bregs, both in phenotype and response to stimuli, and the absence of a definitive single marker (so far) has led to the hypothesis that any B cell can differentiate into a Breg depending on their prevailing environment, rather than a subset derived from a distinct lineage (21). Indeed, signals required for the induction or the promotion of regulatory B cells are the result of an activated inflammatory environment, including pro-inflammatory cytokines, engagement of Toll-like receptors (TLRs) and costimulatory signals (32, 33). This has been reviewed extensively (34). Certainly, evidence from mouse studies show that Bregs are induced in response to inflammation or autoimmunity (13, 35). Moreover, a number of cytokines are involved in promoting Breg responses, many of which have been associated with autoimmune disorders. In autoimmune diabetes a number of cytokines including IL-1β, IL-6 and Interferon (IFN)α, play a role in the development of disease and can contribute to pancreatic β cell death (36). The same cytokines, as well as IL-21, have been shown to activate or expand Breg function (33, 37). IFNα secreted from plasmacytoid DCs (pDCs), in combination with CD40 ligation, can induce IL-10-producing Bregs (37). B cells stimulated with cytosine-phosphate-guanine (CpG) dinucleotides in combination with IL-2, IL-6 and IFNα induced an enhanced IL-10 response (12). Furthermore, IL-1β and IL-6 can drive B cell IL-10-production and Breg differentiation (33). Interestingly, this raises the question of why then in some studies Bregs are numerically or functionally defective in autoimmunity that includes type 1 diabetes (see *Impaired Regulatory B Cell Mechanisms in type 1 diabetes*). One possible reason for this paradox could be explained by other mechanisms required for Breg induction, which are altered in autoimmunity (**Figure 1**).

In a human study of SLE, the failed Breg expansion is attributed to elevated levels of IFNα produced from pDCs during disease, which drives plasmablast differentiation rather than Breg expansion (37). Therefore, it is suggested the concentration levels of cytokine are an important factor in Breg induction, and chronic exposure during inflammation can impair Breg frequency and function (37, 38). Type 1 diabetes, like SLE, is associated with an IFN signature. IFNα expression detected in the pancreatic islets (39) and IFN-associated genes are overexpressed in islets of individuals with type 1 diabetes (40). Additionally, an IFN transcriptional signature has been shown to be increased, even before the onset of human islet autoimmunity (41).

Both IL-21 and CD40 receptor engagement are required for the maturation and function of IL-10-producing B cells, a key study demonstrated in mice (42). Interestingly, naïve B cell responses to IL-21 are diminished in established human type 1 diabetes; however this response is enhanced in pre-diabetic individuals with multiple islet autoantibodies (43). Furthermore, CD4 T follicular helper (Tfh) cells in patients with type 1 diabetes have increased IL-21 production, compared to healthy donors (44, 45).

The importance of CD40: CD40L signaling has been noted in autoimmunity. For example, in autoimmune diabetes, the influence of CD40L blockade on the development of diabetes has been demonstrated in the NOD mouse model (46). This fundamental signaling pathway is important in both T and B cells. In people with type 1 diabetes, CD4^lo^CD40^+^ T cells (T_CD40_) are expanded in peripheral blood (47). In another autoimmune disease, SLE, aberrant expression of CD40L in circulating B cells, in addition to T cells has been noted (48). Furthermore, reduced numbers of CD40^+^ B cells is observed in individuals with type 1 diabetes, compared to healthy donors; however, the levels of CD40 expression on B cells were not measured in this study (28).

Other mechanisms are necessary for the generation or expansion of Bregs and these include both adaptive and innate immune pathways. B cell receptor (BCR) signaling (32, 49), is diminished in B cells from individuals with established type 1 diabetes (25, 43). Signaling through TLR9 changes the frequency and function of IL-10 producing B cells in NOD mice; TLR9 deficiency specifically in B cells increased IL-10 producing cells and protected against diabetes (50). No direct study has demonstrated a mechanism that drives a Breg defect in type 1 diabetes in humans however, and this remains an outstanding question (see *Discussion and Outstanding Questions*).

## Impaired Regulatory B Cell Mechanisms in Type 1 Diabetes

Studies on the numerical and functional defects of Bregs have been described in various autoimmune diseases, including SLE, RA and MS and overall an inverse correlation between the frequency of Bregs and disease activity has been observed (10, 51). It should be noted, however, that studies have also reported either no differences or an increased frequency in these cells between autoimmune individuals and healthy donors (11, 25). Others have demonstrated different levels of CD24^hi^CD38^hi^ Bregs in various autoimmune conditions, compared with healthy controls (52). This theme of contrary results is echoed in studies of type 1 diabetes, which are summarized in **Table 1**.

It still remains unclear whether a defect or an impaired function of Bregs contributes to the development of diabetes or if the observed aberrant frequency and function is a result of chronic inflammation. Studies in the Experimental autoimmune encephalomyelitis (EAE) mouse model of MS has implicated Bregs in disease initiation rather than late-phase progression (53, 54). Moreover, in NOD mice, early treatment (5-6 weeks old) with BCR-activated B cells both delayed and reduced diabetes onset; however later treatment at 9 weeks of age only delayed onset of disease (55). Determining how Bregs contribute to the onset of type 1 diabetes will be of significance when considering immunotherapies targeted at B cells. Future real-time studies of regulatory B cells in islet autoantibody-positive individuals, who have not yet developed overt type 1 diabetes, would improve understanding of this.

### Evidence for Numerical Defects in Regulatory B Cells in Type 1 Diabetes

Specific cell subsets that are associated with regulation, and B cells actively producing IL-10 after *ex vivo* stimulation, have been evaluated to ascertain if Breg frequencies are altered in type 1 diabetes. Whether the frequency of Breg-associated populations are altered, which include CD5^+^CD1d^hi^ and transitional CD24^hi^CD38^hi^ B cells, has been inconclusive when comparing patients to healthy donors (**Table 1**). A likely contributor to the disparity in these studies is the different sets of immune markers used to distinguish discrete populations or analysis of different Breg subsets. Use of an increased number of immune markers and high-dimensional profiling will help to determine more discrete B cell subsets and may resolve these dichotomies. For example, detailed characterisation has shown that human B cells which readily produce IL-10 are enriched in both T2 (CD27^-^IgM^+^IgD^+^) and CD27^+^ B cells in the transitional CD24^hi^CD38^hi^ compartment (52). Furthermore, stimulation *via* TLR9 resulted in enhanced IL-10 expression in the transitional T3 subset (52).

Direct assessment and evaluation of IL-10 production from B cells requires exogenous stimuli. Targets include either the innate TLRs or other receptors such CD40 or the BCR, either separately or by co-engagement, and if IL-10 is measured by intracytoplasmic staining, the addition of PMA/Ionomycin is also required (56). So far in type 1 diabetes, the studies employing CD40L and TLR stimulants - either LPS [TLR4] or CpG [TLR9] in culture before assessment, or with PMA/ionomycin alone - have shown a decrease in numerical frequency of IL-10-producing B cells from peripheral blood samples (28–30). However, when a combination of LPS and CPG was used, with the addition of IL-21, which can drive IL-10 production from B cells (42), the investigators found no difference in IL-10^+^ B cells, in either naïve or memory compartments (27). A detailed summary of these studies is described in **Table 1**. It is clear that both the stimulation conditions and the appropriate markers to identify distinct populations are necessary for a more accurate overview on how B cell subsets are altered in type 1 diabetes.

In addition, a key disparity between studies is how accurately healthy donors were age-matched (**Table 1**). It is clear that subsets, such as transitional CD24^hi^CD38^hi^ B cells, enriched with IL-10^+^ B cells, decline with age (27, 57), which is an important note for future studies. Recently, in children with type 1 diabetes, a decrease in both the CD24^hi^CD27^+^ (B10) and transitional CD24^hi^CD38^hi^ IL-10^+^ B cells but not in CD38^hi^CD27^+^IL-10^+^ plasmablasts was found (31). This numerical decrease was also negatively correlated with HbA1c levels (31), as was the frequency of CD24^hi^CD38^hi^ B cells in a study by Wang et al. (30). In view of the recent observation that the frequency of pancreatic CD20^+^ B cells correlates with earlier diagnosis of a rapidly progressing and more aggressive disease (58), considering both age and clinical parameters in studies assessing regulatory B cells will be particularly important.

Currently, very few studies have assessed Breg populations in individuals with multiple islet autoantibodies who are classed as ‘at risk’ or in ‘stage 1’ or ‘stage 2’ (59) of developing diabetes. Kleffel et al. reported that individuals with multiple islet autoantibodies (like individuals with diabetes) had significantly fewer IL-10^+^ B cells, compared to healthy controls (28). However, Saxena et al. observed that antibody positive individuals had increased CD5^+^IL-10^+^ B cells, compared to both healthy and diabetic controls (29). Overall, whether numerical differences exist in IL-10-producing B cells in individuals with islet autoantibodies remains a key outstanding question, which needs to be addressed in order to refine and improve immunotherapy targeted at B cells.

Although evidence has been provided in mouse models that IL-10^+^ B cells can control autoimmune diabetes (55), few studies have addressed the number of IL-10-producing B cells in mice that have developed overt disease. Recent work from our group has demonstrated that NOD mice that developed diabetes showed a reduced splenic IL-10^+^ B cell population, measured by intracytoplasmic staining, compared to mice that were long-term normoglycemic or ‘naturally-protected’ from diabetes (>35 weeks old) (60). Also, the frequency of IL-10^+^ B cells was dependent on the B cell stimulation used, with anti-CD40 ligation highlighting the greatest loss in frequency of IL-10^+^ B cells in diabetic NOD mice (60). This again focuses our attention on the need for better understanding and a more comprehensive use of different, combined stimuli. Additionally, we observed either no difference or increased IL-10 secretion in the mice that had developed diabetes, dependent on the stimulus used for study of the B cells (60). To date, type 1 diabetes studies reporting differences in IL-10^+^ B cells have not evaluated IL-10 secretion. Increased IL-10^+^ B cell frequency has been demonstrated in long-term normoglycemic or ‘naturally protected’ NOD mice in pancreatic islets (28, 61), suggesting a Breg-mediated protection against β cell destruction. For further discussion of Bregs related to pancreatic islets see *Regulatory B cells in Pancreatic Islets*.

### Impaired Regulatory B Cell Function in Type 1 Diabetes

Functional studies in Bregs have described numerous immunosuppressive mechanisms of IL-10-producing B cells, including inhibiting pro-inflammatory cytokines from immune cells and promoting regulatory T cell differentiation (10, 51, 62), together with dampening of antigen presenting cell (APC) responses (11, 12). In autoimmune conditions, failed mechanisms of Breg immunosuppression are observed. In SLE patients, B cells fail to produce IL-10 in response to CD40 ligation and are unsuccessful in suppressing Th1 responses (10). CD24^hi^CD38^hi^ Bregs from individuals with active RA are unable to convert CD4^+^CD25^-^ into Tregs or suppress Th17 responses (51). Moreover, CD19^+^CD27^+^IL-10^+^ B cells from donors with RA fail to suppress IFNγ from CD4^+^ T cells, compared to healthy individuals (62).

Evidence for diminished Breg function in human type 1 diabetes studies is limited. A recent study demonstrated that a numerical deficiency of Bregs was coupled with a functional defect in patients (30). Here, IL-10-producing B cells in healthy volunteers were enriched in the CD24^hi^CD38^hi^ transitional subset, after CD40L and CPG stimulation, as shown previously (10). Furthermore, CD24^hi^CD38^hi^ B cells inhibited effector cytokines from CD4^+^ T cells and promoted CD4^+^FoxP3^+^ Tregs, in an IL-10-dependent manner (10). However, in patients with type 1 diabetes, CD24^hi^CD38^hi^ B cells failed to reduce IFNγ, TNFα and IL-17 production from CD4^+^ T cells (30). Conversely, Kleffel et al. showed that expanded IL-10-producing B cells from individuals with type 1 diabetes could suppress IFNγ production in PBMC cultures, in the presence of IA-2 peptide (28). However, the generation of IL-10^+^ B cells from both individuals with type 1 diabetes and those with multiple islet autoantibodies was significantly impaired compared to healthy donors (28).

Murine studies have illustrated how regulatory B cells can control autoimmunity (8). Research has focused on how B cells can suppress autoimmune diabetes, demonstrating a role for IL-10-independent (63) and IL-10-dependent (55) mechanisms of B cell-mediated immunosuppression. However, data describing impaired regulatory B cell responses in mice, NOD or otherwise, are limited. TLR4-activated B cells from NOD mice that have developed diabetes suppress insulin-specific CD8 T cells, and in a B cell: DC : CD8 T cell co-culture produced significant amounts of IL-10 (60). This required the presence of the pathogenic CD8 T cells, because without pathogenic CD8 T cells in the cultures, the TLR4-induced B cells produced significantly less IL-10 and were less efficient in reducing DC activation. We also showed, in NOD mice with established diabetes, that CD40-ligation on B cells, followed by co-culture with DCs, the ability to reduce DC activation was decreased and resulted in a contact-dependent increase in IFNγ secretion, compared to NOD mice naturally-protected from autoimmune diabetes (60). In line with these observations, B cells from hyperglycemic NOD mice adoptively transferred into B cell-depleted long-term normoglycemic NOD animals promoted diabetes onset (28).

Other mechanisms of Breg suppression, independent of IL-10 expression and dependent on cell-contact have been noted. For example, PD-L1 and FasL exert suppression *via* apoptosis of target cells upon engagement with their receptors (2, 64). B cells that express FasL can induce apoptosis and suppress proliferation of CD4^+^ T cells (3, 65). In mice, FasL can be induced by TLR4 activation in CD5^+^CD1d^+^ Bregs (65) and in the NOD mouse model can be activated with LPS (TLR4), resulting in TGFβ production, which inhibits Th1 responses and diabetes progression (63). In humans FasL^hi^CD5^+^ B cells are increased in frequency in individuals with type 1 diabetes, compared to both islet autoantibody positive and healthy donors (29), although here the levels of TGFβ production with stimulation was not assessed. Interestingly, in this study the frequency of CD5^+^IL-10^+^ B cells did not differ between healthy and diabetes donors (described in **Table 1**), but the percentage of Fas-expressing CD5^+^IL-10^+^ B cells was elevated in donors with type 1 diabetes (29). This is indicative of Fas-FasL B cell interplay, with elevated CD5^+^FasL B cells targeting more apoptosis-sensitive CD5^+^IL-10^+^ B cells, which results in fewer IL-10^+^ B cells in individuals with autoimmune diabetes (29) (**Figure 1**, red box).

It remains inconclusive if there is an intrinsic developmental Breg defect that contributes to disease progression in individuals that develop type 1 diabetes, and is complicated by the lack of a definitive Breg marker and their heterogeneity. It is possible the differences in Bregs observed in some studies (described in **Table 1**) results from the inflammatory environment that occurs with the progression of disease, which indirectly impacts the size or function of the Breg compartment (see **Figure 1**). Indeed, IL-10-producing B cells are expanded in mice predisposed to autoimmunity, compared to non-susceptible mice (32). Furthermore, IL-10^+^ splenic B cells are expanded in 4-week-old NOD mice and IL-10^+^ B cells from normoglycemic NOD mice are still capable of suppressing T cell-mediated diabetes (28).

Overall, these studies described above in type 1 diabetes suggest that further interrogation is warranted on the defective or dysfunctional Bregs observed, including the autocrine B cell mechanisms and crosstalk with other immune cells (see *Discussion and Outstanding Questions*). Further studies, using both human peripheral blood and tissue sites in different cohorts, taking into account that IL-10^+^ B cell immune-phenotypes are variable with age (66), will provide insight into Breg defects.

## Regulatory B Cells in Pancreatic Islets

B cells residing in pancreatic islets during inflammation contribute to the destruction of β cells, and consequently a loss in the secretion of insulin. Evidence for this direct pathogenic role has been shown by B cell depletion studies in the NOD mouse model, highlighting a reduction in effector T cell function inhibiting tissue-specific inflammation in treated mice (67, 68). In NOD mice, B-1a cells located in the pancreas, early in diabetes, play a role in initiation of disease (69). Furthermore, the observation of different profiles of insulitis in human pancreatic islets, with increased frequency of CD20 B cells correlate with a more progressive earlier diagnosis (58).

Previously, we have alluded to proposed interactions between regulatory B cells and the inflammatory pancreatic islet environment, and how Bregs can control inflammation (70). Islet-specific B cells in naturally-protected normoglycemic NOD mice have increased IL-10 and CD40 expression (28). More recently, we have corroborated this work and demonstrated B cells from naturally-protected NOD mice have an increased frequency of B cells expressing IL-10, CD80 and CD40 (61). In this study we also described an enrichment of CD19^int^CD138^hi^CD44^hi^Ki67^+^ dividing plasmablasts in naturally-protected NOD mice (61) a phenotype attributed to IL-10 production (12). Alongside this increase in regulatory B cells, a significant increase of CTLA4^+^FoxP3^+^ Tregs was also observed (61), possibly indicating some Breg-Treg crosstalk, which suppresses local pancreatic inflammation. However, it is unknown if this crosstalk is dependent on the expression of IL-10. It is possible that other IL-10-independent Treg induction by B cells may occur, as shown by the requirement for Breg expression of GITR ligand (71). Moreover, it is currently unclear if the altered pancreatic milieu in naturally-protected NOD mice is responsible for the induction of these regulatory immune cells, or a result of expanded IL-10^+^B cells in the periphery (60). IL-10^+^ B cells can be detected in the pancreatic islets of younger NOD mice after CD40 ligation along with a PMA/Ionomycin stimulation, albeit the frequency of IL-10^+^ B cells was very low (28, 61), and it is unknown if they have any role in controlling local β cell damage *in vivo*.

## Discussion and Outstanding Questions

As discussed above, studies of regulatory B cells in type 1 diabetes are limited in comparison to other autoimmune diseases that include SLE, RA and MS, and thus lessons can be learned in order to extrapolate the findings to direct key research in type 1 diabetes. Finally, we discuss future and outstanding research questions that will advance the treatments of type 1 diabetes.

1. A deeper understanding of the different Breg repertoires that are IL-10-producing or IL-10-competent, together with the altered frequency and function in different stages of autoimmune diabetes development.

The complex picture described, so far, in diabetes and other autoimmune diseases may reflect the divergent role of Breg subsets in various disease settings. Different subsets of Bregs, based on their maturity, may be more influenced by the level of inflammation and disease stage of the individual. As previously shown, different immune profiles for B cell and T cell responses are dependent on disease stage or progression (43). It should also be noted that IL-10-producing B cells can also secrete TNF and IL-6 and so there is heterogeneity in Breg cytokine production (72).

2. Breg interplay and crosstalk with both other B cell populations and different immune cells to dissect the relationships that impact frequency and function (**Figure 1**).

Interrogating Breg: immune cell crosstalk will uncover aberrant regulatory feedback loops. Cell subsets like pDCs (37) or other DC subsets will reveal how Bregs dampen, or fail to dampen APCs. Other Breg studies highlight immunosuppressive mechanisms *via* invariant NKT cells dependent on the surface molecule CD1d (73). Other studies describe a feedback loop between T cells and B cells, *via* CD40:CD40L interactions, to develop regulatory function, which differentially regulate T cell proliferation and Th1 responses (74).

3. Determine the impact of defective Breg frequency and function. Do impaired Bregs contribute to diabetes initiation or progression or both?

Determining if the defect in Breg frequency and/or function is a consequence of chronic inflammation or a contributor to the development of diabetes will have an impact on how B cell depletion therapy is exploited in individuals during various stages of disease progression. Furthermore, understanding if impaired Bregs contribute to disease due to the lack of immunosuppressive action or if Breg plasticity results in a further progression of disease under certain chronic conditions should be addressed.

4. The use of immunotherapies to either selectively expand Bregs or target pathogenic B cells but spare regulatory B cells.

So far, only a pan-B cell depletion approach has been trialed in type 1 diabetes (Rituximab) (7), and therefore we can only discuss preclinical studies that approach expanding Bregs *in vivo* or targeting a specific B cell population. Expansion of CD73^+^ regulatory B cells after treatment with a small molecule inhibitor that disrupts the *Aicda*-encoded activation-induced cytidine deaminase protein (AID) results in the inhibition of diabetes development in the NOD mouse (75). Conversely, AID deficiency in the NOD mouse model can accelerate type 1 diabetes development (76) and therefore the role of AID in diabetes progression requires further investigation. An additional B cell-targeted therapeutic approach is to selectively deplete effector B cells preserving regulatory B cells; however this is complicated by the lack of a definitive Breg marker. Interestingly, targeting of B cells *via* the blockade of the B cell activating factor (BAFF) induced an increase of IL-10^+^ B cells and diabetes protection (77). Furthermore, in this study, anti-CD20 treatment depleted this IL-10-producing B cell population, suggesting that Bregs are more sensitive to deletion during anti-CD20 treatment (77). This Breg sensitivity may have contributed to the limited success of the Rituximab clinical trial (7). However, as discussed above, a deeper understanding of Bregs during the development of type 1 diabetes is needed to harness and develop successful B cell targeted immunotherapies.

Overall, the pathogenesis of type 1 diabetes is complex and multi-stage, and requires a number of pathogenic cell types that give rise to the development of disease. Equally, it is clear that balanced against these pathogenic cells are regulatory cells, that include both T and B cell subsets. Defining the roles of these less-understood Breg subsets will provide important information to be further studied in humans with the aim of increasing therapeutic opportunities.

## Author Contributions

JB wrote and edited the manuscript. FSW edited the manuscript. All authors contributed to the article and approved the submitted version.

## Funding

JB is supported by an Independent Fellowship funded by Research England’s Expanding Excellence in England (E3) fund via EXCEED. Medical Research Council (UK) grant MR/K021141/1 was awarded to FSW.

## Conflict of Interest

The authors declare that the research was conducted in the absence of any commercial or financial relationships that could be construed as a potential conflict of interest.

## Publisher’s Note

All claims expressed in this article are solely those of the authors and do not necessarily represent those of their affiliated organizations, or those of the publisher, the editors and the reviewers. Any product that may be evaluated in this article, or claim that may be made by its manufacturer, is not guaranteed or endorsed by the publisher.
